# Fish processing wastes for microbial enzyme production: a review

**DOI:** 10.1007/s13205-012-0099-8

**Published:** 2012-10-30

**Authors:** Faouzi Ben Rebah, Nabil Miled

**Affiliations:** Laboratoire de Biochimie et de Génie Enzymatique des Lipases, ENIS, BPW 1173-3038 Sfax, Tunisia

**Keywords:** Fish waste, Growth media, Protease, Lipase, Chitinolytic enzymes, Ligninolytic enzymes

## Abstract

Fishery processing industries generate large amounts of by-products. The disposal of these wastes represents an increasing environmental and health problem. To avoid wasting these by-products, various disposal methods have been applied including, ensilation, fermentation, hydrolysate and fish oil production. Interestingly, fish by-products provide an excellent nutrient source for microbial growth useful in enzyme production process, which is largely governed by the cost related to the growth media. Fish wastes (heads, viscera, chitinous material, wastewater, etc.) were prepared and tested as growth substrates for microbial enzymes production such as protease, lipase, chitinolytic and ligninolytic enzymes. This new approach described in this review can reduce environmental problems associated with waste disposal and, simultaneously, lower the cost of microbial enzyme production.

## Introduction

In recent years, there has been a constant increase in the exploitation of fish resources and the estimated quantity used for human consumption (105.6 million tons) is globally 75 % of the worldwide fish production. The remaining 25 % of the catch (34.8 million tons) are considered as wastes (FAO 2007). Furthermore, the commercial fish processing industry generates large quantities of solid waste and wastewater. Solid waste which represents 20–60 % of the initial raw material contains various kinds of residues (whole waste fish, fish head, viscera, skin, bones, blood, frame liver, gonads, guts, some muscle tissue, etc.) (Awarenet 2004). In some countries, these discards are not utilized, but incinerated or dumped at sea causing environmental problems (Bozzano and Sarda 2002). Recently, environmental regulations are becoming stricter, requiring new disposal methods based on the fact that fish wastes (solid waste and wastewater) may considered as an important source of protein, lipids and minerals with high biological value (Toppe et al. 2007; Kacem et al. 2011).

To avoid wasting by-products, various conventional disposal methods have been applied including ensilation and fermentation for the production of high-protein meals for animal feeds (Faid et al. 1997; Hassan and Heath 1986) as well as composting (Liao et al. 1997). Recent advances in industrial biotechnology process are exploited for an economic utilization of wastes in producing higher added value products. For example, fish oil with higher level of polyunsaturated fatty acids beneficial for human health (Kim et al. 2006; Zampolli et al. 2006; Chen et al. 2006) was produced and integrated into food products and beverages (Rubio-Rodriguez et al. 2010). Moreover, fish skin or cartilage from some species could be excellent raw materials for producing gelatin or chondroitin sulphate useful in food, cosmetic and pharmaceutical sectors (Blanco et al. 2006; Karim and Bhat 2009). By-products can be also hydrolysed by applying various treatments (heat, enzymatic and chemical treatments) and the hydrolysate quality can be also improved by fermentation (Yamamoto et al. 2005; Yano et al. 2008; Xu et al. 2008). Generally, the obtained hydrolysates may have biological and functional properties of interest in various sectors (human nutrition, cosmetology, aquaculture, microbiology, etc.) (Ben Rebah et al. 2008; Liaset et al. 2000; Martone et al. 2005). For example, proteolytic hydrolyses were reported to produce peptides with interesting biological activities (anti-hypertensers, immuno-modulators, antioxidants, anticoagulants, etc.) useful in the treatment of several diseases (osteoporosis, arthritis, diabetes, obesity, etc.) (Kim and Mendis 2006).

Interestingly, fish by-products provide an excellent source for microbial growth, which can be exploited in producing various metabolites (lysine, enzymes, etc.) (Coello et al. 2000; Vazquez et al. 2006, 2008). A large number of microorganisms including bacteria, yeast and fungi produce different groups of enzymes (protease, lipase, etc.) having high biotechnological interest (food processing, detergent, textile, pharmaceutical products, medical therapy, etc.) (Underkofler et al. 1958). Microbial synthesis of enzymes has been reported to be influenced by various factors such as carbon sources, nitrogen sources, and operating parameters (temperature, pH, etc.) (Frost and Moss 1987; Gupta et al. 2004; Jacob and Prema 2006; Palaniyappan et al. 2009). Generally, agro-industrial residues mainly composed of complex polysaccharides are considered the best substrates for microbial growth and enzyme production (Pandey et al. 1999). The economies of this process are governed mostly by the cost and availability of a suitable carbon and nitrogen sources. Therefore, the investigation of low cost substrates easily available such as fish processing wastes became a necessity opening new avenues for their effective production and utilization. In this review, we will present the potential of different fish processing by-products tested as growth substrates for microbial enzyme production.

## Proteases

Proteases are one of the most important groups of industrial enzymes, representing more than 65 % of the of global industrial enzyme market (Banik and Prakash 2004). Various microorganisms have been used to produce these enzymes (*Penicillium* sp. *Serratia marcescens*, *Streptomyces* sp., *Rhizopus oryzae*, *Pseudomonas*, *Bacillus* sp., *Vibrio*, etc.) (Banerjee et al. 1993; De Azeredo et al. 2004; Joo and Chang 2005; Vazquez et al. 2006).

Because of their vast diversity and their specificity of action, proteases have a large variety of biotechnological applications (Kumar and Takagi 1999; Gupta et al. 2002). Their use involves different industrial sectors, such as the detergent industries (Banik and Prakash 2004), bioremediation processes (Roberts et al. 2007), leather industry (Taylor et al. 1987), bioprocessing of used X-ray films for silver recovery (Fujiwara and Yamamoto 1987) pharmaceutical industries and protein hydrolysate production (Banik and Prakash 2004). Recently, the application of protease in producing bioactive peptide has received great attention as a viable alternative to chemical approach (Suetsuna 2000; Ma et al. 2007).

Numerous investigators have looked for ways of producing microbial proteases using inexpensive media. Interestingly, it was demonstrated that fish processing wastes (fish meat wastes, chitinous material from cephalopods and wastewater) offer good potential for this purpose (Triki-Ellouz et al. 2003; Vazquez et al. 2006; Wang and Yeh 2006; Haddar et al. 2010).

Fish processing by-products including heads and viscera were utilized for preparing microbial growth media. In some cases, fish wastes were cooked, pressed, minced and then dried (80 °C for 24–48 h) in order to obtain fish powder. In other cases, wastes were boiled in water and supernatants were recuperated. Moreover, protein hydrolysates obtained by acid, alkali, or enzymatic treatments of raw or defatted by-products were also used as a nitrogen source for protease production (Coello et al. 2000; Triki-Ellouz et al. 2003). Generally, fish powder or supernatants were added to the microbial basal medium (Table 1). For example, *Pseudomonas aeruginosa* MN7 and *Bacillus subtilis* were cultivated in media containing combined heads and viscera powder allowing an acceptable level of protease production (Ellouz et al. 2001; Triki-Ellouz et al. 2003). Similarly, marine peptones obtained from fish viscera of various marine species (rainbow trout “*Oncorhynchus mykiss*”, swordfish “*Xiphias gladius*”, squid “*Loligo vulgaris*” and yellowfin tuna “*Thunnus albacares*”) allowed higher levels of protease activity for two vibrio species (*Vibrio anguillarum* and *Vibrio splendidus*) while compared to basal medium containing commercial peptones (Vazquez et al. 2006). According to Esakkiraj et al. (2009), a defatted tuna waste allowed higher level of protease activity by *Bacillus cereus* (134.57 U/ml) while compared to acid and alkali hydrolysates (60.37 and 65.96 U/ml, respectively) and to commercial peptone (124.90 U/ml). The enhancement of protease production in the defatted fish-based medium may be attributed to the lipid-free nature of product, which could support the protease synthesis by the microbial species than other nitrogen source preparations. A similar observation was notified for lipase production by *Rhizopus oryzae* (Ghorbel et al. 2005). More recently, a significant improvement of the alkaline protease production by *Bacillus mojavensis* A21 was obtained using *Sardinella* peptone (Haddar et al. 2010). However, is very important to note that optimal protease production is controlled by several growth conditions such as the medium composition (carbon and nitrogen sources, mineral salts, etc.), agitation, culture temperature and initial medium, pH, etc.

**Table 1:** Protease production by various microbial strains grown in fish processing waste based media

Fish raw materials	Preparation of the growth media^a^	Microbial strains	Activity (U/ml)	References
Heads and viscera of *Sardinella*	Raw materials cooked, pressed, minced and dried (80 °C, 24–48 h)	*Pseudomonas aeruginosa* MN7	7,800	Triki-Ellouz et al. (2003)
Heads and viscera of *Sardinella*	Raw materials cooked, pressed, minced and dried (80 °C, 24–48 h)	*Bacillus subtilis*	720	Ellouz et al. (2001)
Viscera from rainbow trout, swordfish, squid and yellowfin tuna	Raw materials ground with water and supernatants recovered by centrifugation and used to prepare the peptone	*Vibrio anguillarum*	35–68	Vazquez et al. (2006)
Viscera from rainbow trout, swordfish, squid and yellowfin tuna	Raw materials were ground with water and supernatants recovered by centrifugation and used to prepare the peptone	*Vibrio splendidus*	9–30	Vazquez et al. (2006)
Raw tuna waste	Raw materials cooked, bones removed, pressed to remove water and fat, pressed, minced and dried (80 °C, 24–48 h)	*Bacillus cereus*	74.77	Esakkiraj et al. (2009)
Defatted tuna waste	Extraction with chloroform/methanol	*Bacillus cereus*	134.57	Esakkiraj et al. (2009)
Acid-hydrolyzed tuna waste	Method described by Gao et al. (2006)	*Bacillus cereus*	60.37	Esakkiraj et al. (2009)
Alkali-hydrolyzed tuna waste	Method described by Batista (1999)	*Bacillus cereus*	65.96	Esakkiraj et al. (2009)

^a^The obtained fish powder was added to the basal medium

Several microbial strains grown in basal medium supplemented with different chitinous material preparations [shrimp shell powder (SSP), squid pen powder (SPP), chitin flake of shrimp shell (CFSS), chitin flake of crab shell (CFCS), shrimp and crab shell powder (SCSP)] (Table 2) were tested for protease activity. Generally, chitinous materials collected from marine food processing industry were treated (dried, milled, and sieved to powder with diameters of <0.053 mm) and added to the basal medium at different proportions as reported by Wang et al. (2002a). According to Table 2, protease activity was affected by the nature of the strain and the used carbon/nitrogen sources. For example, for both *Bacillus subtilis* TKU007 and *Bacillus* sp. TKU004, SSP was more suitable for protease production (Wang and Yeh 2006) than the other carbon/nitrogen sources. However, for the production of protease by *L. paracasei* subsp. *paracasei* TKU010, SPP (0.12 U/ml) was more suitable than SSP (0.08 U/ml) (Wang et al. 2008a). Also, in the case of *Serratia ureilytica* TKU013, SPP (0.200 U/ml) was more suitable than SSP (0.190 U/ml) (Wang et al. 2009a). According to Wang et al. (2008b), higher protease activity might be controlled by the ratio of protein and chitin. In the findings of Wang et al. (2008b), the ratio of protein and chitin (48:38) in SSP is much closer than that of SPP (61:38) allowing a protease activity by *Chryseobacterium* sp. TKU014 of 0.010 and 0.007 U/ml with SSP and SPP, respectively. In addition to that, mineral salts may have a suppressed role on the protease productivity as reported for *L. paracasei* subsp. *paracasei* TKU010 and TKU012 **(**Wang et al. 2008a).

**Table 2:** Protease production by various microbial strains grown in chitinous material based media

Chitinous materials (rate added to the basal medium in % w/v)	Microbial strains	Protease activity (U/ml)	References
Shrimp shell powder (SSP)^a^			
1	*Chryseobacterium* sp. TKU014	0.010	Wang et al. (2008b)
1.5	*Bacillus subtilis* TKU007	0.350	Wang and Yeh (2006)
1	*Bacillus subtilis* TKU007	0.350	Wang and Yeh (2006)
1	*Bacillus* sp. TKU004	0.090	Wang et al. (2006a)
1	*Lactobacillus paracasei* subsp *paracasei* TKU010	0.080	Wang et al. (2008a)
1	*Lactobacillus paracasei* subsp *paracasei* TKU012	0.130	Wang et al. (2008c)
1.5	*Serratia ureilytica* TKU013	0.190	Wang et al. (2009a)
2	*Bacillus cereus* TKU006	2.070	Wang et al. (2009b)
1	*Serratia* sp. TKU016	0.160	Wang et al. (2010)
Squid pen powder (SPP)^a^			
2	Bacillus sp. TKU004.	0.065	Wang et al. (2006a)
1	*Chryseobacterium* sp. TKU014	0.007	Wang et al. (2008b)
2	*Bacillus subtilis* TKU007	0.220	Wang and Yeh (2006)
1	*Bacillus subtilis* TKU007	0.220	Wang and Yeh (2006)
1	*Bacillus* sp. TKU004	0.060	Wang et al. (2006a)
1	*Lactobacillus paracasei* subsp *paracasei* TKU010	0.120	Wang et al. (2008a)
1	*Lactobacillus paracasei* subsp *paracasei* TKU012	0.140	Wang et al. (2008c)
1.5	*Serratia ureilytica* TKU013	0.200	Wang et al. (2009a)
1	*Serratia* sp. TKU016	0.090	Wang et al. (2010)
Chitin flake of shrimp shell (CFSS)			
2	*Bacillus subtilis* TKU007	<0.010	Wang and Yeh (2006)
1	*Bacillus subtilis* TKU007	<0.010	Wang and Yeh (2006)
1	*Bacillus* sp. TKU004	<0.010	Wang et al. (2006a)
1	*Lactobacillus paracasei* subsp *paracasei* TKU010	<0.010	Wang et al. (2008a)
1	*Lactobacillus paracasei* subsp *paracasei* TKU012	<0.010	Wang et al. (2008c)
Chitin flake of crab shell (CFCS)			
2	*Bacillus subtilis* TKU007	<0.010	Wang and Yeh (2006)
1	*Bacillus subtilis* TKU007	<0.010	Wang and Yeh (2006)
1	*Bacillus* sp. TKU004	<0.010	Wang et al. (2006a)
1	*Lactobacillus paracasei* subsp *paracasei* TKU010	<0.010	Wang et al. (2008a)
1	*Lactobacillus paracasei* subsp *paracasei* TKU012	<0.010	Wang et al. (2008c)
Shrimp and crab shell powder (SCSP)			
1	*Bacillus subtilis* TKU007	nd	Wang and Yeh (2006)
1	*Bacillus* sp. TKU004	0.060	Wang et al. (2006a)
1	*Lactobacillus paracasei* subsp *paracasei* TKU012	0.090	Wang et al. (2008c)
1	*Lactobacillus paracasei* subsp *paracasei* TKU010	0.040	Wang et al. (2008a)

*nd* not detectable

^a^Dried materials of SPP, SSP were prepared as described earlier (Wang et al. 2002a)

In order to enhance the protease production, shrimp and crab shell powder were treated using chemical reagents (HCl, NaOH and HCl/NaOH (Oh et al. 2000) and used for microbial growth. The chemical treatments and the rate of the preparation added to the microbial basal medium affect considerably the time of growth and the protease activity as reported by Liang et al. (2006) for *Monascus purpureus* CCRC31499. Interestingly, protease produced under optimal culture conditions can be exploited in the deproteinization of shrimp and crab shell wastes. For example, it was demonstrated that protease of *P. aeruginosa* K-187 allowed higher protein removal from crustacean wastes. The removal rates were about 72 % for shrimp and crab shell powder, 78 % for natural shrimp shell and 45 % for acid-treated shrimp and crab shell powder (Oh et al. 2000).

Wastewater from fish processing industry supplemented with cuttlefish by-products powder was also tested as growth media for microbial growth and protease production by five bacterial species (*Bacillus licheniformis*, *Bacillus subtilis*, *Pseudomonas aeruginosa*, *Bacillus cereus* BG1, and *Vibrio parahaemolyticus*). According to Souissi et al. (2008), all the tested strains can obtain their carbon and nitrogen source requirements directly from proteins of the cuttlefish by-products and, interestingly, the addition of the fishery wastewater improved the protease activity. For example, the protease activity of *Bacillus cereus* BG1, increased from 467 U/ml to reach 2771 U/ml while adding fishery wastewater to the cuttlefish by-product-based medium (Table 3). The positive effect on protease activity may be explained by the presence of very specific growth factors and amino-acids in the fishery wastewater.

**Table 3:** Proteases produced by some microbial strains cultivated in fishery waste based media (Souissi et al. 2008)

Strains	Type of protease	Growth media	Protease activity (U/ml)	Time of growth (h)
*Bacillus cereus* BG1	Metalloprotease	M^a^	487	48
		M + FWW^b^	756–2,771	48
*Bacillus subtilis*	Serine protease	CF^c^	178	16
		CF + FWW^d^	0–392	16
*Bacillus licheniformis*	Serine protease	CF	407	24
		CF + FWW	138–821	24
*Pseudomonas aeruginosa*	Metalloprotease	CF	1,680	24
		CF + FWW	160–1,694	24
*Vibrio parahaemolyticus*	Serine protease	CF	1,607	24
		CF + FWW	196–2,487	24

^a^M: 10 g /l of maltose and 10 g /l of cuttlefish by-products powder in artificial sea water (Krieg and Holt 1984)

^b^M + FWW: M mixed with FWW (fishery wastewater) at different concentrations

^c^CF: cuttlefish by-product medium (g /l of cuttlefish by-products powder in distilled water; Cuttlefish by-products powder was prepared from guts and stomachs (rinsed, heated, pressed, then, minced and dried at 80 °C for 24–48 h as reported by Souissi et al. 2008

^d^CF + FWW medium: CF prepared in crude and diluted FWW

The diversified utilization of fish processing wastes as potential media for microbial proteases production is expected to deliver an attractive and promising strategy for enzyme large-scale production. In commercial practice, the optimization of medium composition is done to maintain a balance between the various microbial growth nutrients. However, no defined medium has been established for the best production of proteases from different microbial sources. Generally, each microbial strain has its own special conditions for maximum enzyme production. Therefore, experiments should be conducted to elucidate the behaviour of each strain with each fish waste to identify inducers present in the fish waste. These researches would provide the incentive for commercial developments leading to large-scale and cost-effective production of proteases.

## Lipases

Lipases are a class of enzymes which catalyse the hydrolysis of long chain triglycerides and are of considerable commercial interest in various industrial applications (detergent, food, flavour industry, biocatalytic resolution of pharmaceuticals, esters and amino acid derivatives, fine chemicals, agrochemicals, use as biosensor, bioremediation, cosmetics, perfumery, etc.) (Hasan et al. 2006). With the rapid development of enzyme technology, lipases are currently receiving much attention involving various selected microorganisms especially from fungi, bacteria, and yeasts (Sharma et al. 2001).

Numerous investigators have looked for ways of producing microbial lipases using low cost media. A variety of fish processing by-products contain growth factors offering good potential as culture media, as shown by the high level of lipase activity produced by some microbial strains (Table 4). As reported in Table 4, various pre-treatment processes (heat treatment, chemical and enzymatic treatment, etc.) were applied on fish wastes before being used as growth media (Ben Rebah et al. 2008; Esakkiraj et al. 2010a). For example, lipase activity of *Staphylococcus xylosus* was evaluated in supernatants generated from boiled fish wastes (tuna, sardine, cuttlefish, and shrimp by-products) supplemented or not with the basal growth medium (containing 17 g/l casein peptone, 5 g/l yeast extract and 2.5 g/l glucose). Depending on the waste origin and the proportion of the added basal medium, lipase activity varied much among the tested samples and supernatants generated from shrimp and cuttlefish by-products exhibited the highest lipase activity (28 U/ml) (Ben Rebah et al. 2008). Similar experiments were reported while growing *Staphylococcus epidermidis* CMST Pi 2 (isolated from the intestine of shrimp *Penaeus indicus*) in a medium containing raw and treated (defatted, alkali and acid hydrolysates) tuna by-products (Esakkiraj et al. 2010a).

**Table 4:** Production of lipase by different microbial species grown in fish processing by-products

Fish raw materials	Preparation of the growth media	Microbial strains	Lipase activity (U/ml)	References
Defatted tuna by-products	Extraction with chloroform/methanol^a^	*Staphylococcus epidermidis* CMST Pi 2	12.63	Esakkiraj et al. (2010a)
Defatted tuna by-products	Extraction with chloroform/methanol^a^	*Staphylococcus epidermidis* CMST Pi 2	14.20	Esakkiraj et al. (2010a)
Tuna by-products	Raw materials were cooked, bones were removed, pressed to remove water and fat, pressed, minced and dried (80 °C, 24–48 h)^a^	*Staphylococcus epidermidis* CMST Pi 2	8.17	Esakkiraj et al. (2010a)
Acid-hydrolyzed tuna waste	Method described by Gao et al. (2006)^a^	*Staphylococcus epidermidis* CMST Pi 2	8.03	Esakkiraj et al. (2010a)
Alkali-hydrolyzed tuna waste	Method described by Batista (1999)^a^	*Staphylococcus epidermidis* CMST Pi 2	8.13	Esakkiraj et al. (2010a)
Shrimp by-products	Raw materials were boiled (100 °C for 20 min) in water and supernatants were recuperated by centrifugation^b^	*Staphylococcus xylosus*	19–28	Ben Rebah et al. (2008)
Cuttlefish by-products	Raw materials were boiled (100 °C for 20 min) in water and supernatants were recuperated by centrifugation^b^	*Staphylococcus xylosus*	5–9.50	Ben Rebah et al. (2008)
Tuna by-products	Raw materials were boiled (100 °C for 20 min) in water and supernatants were recuperated by centrifugation^b^	*Staphylococcus xylosus*	0–4	Ben Rebah et al. (2008)
Sardine by-products	Raw materials were boiled (100 °C for 20 min) in water and supernatants were recuperated by centrifugation^b^	*Staphylococcus xylosus*	0–3	Ben Rebah et al. (2008)
Cod liver oil	1 % of fish oil added to the basal medium	*Staphylococcus epidermidis* CMST Pi 1	14.8	Esakkiraj et al. (2010b)

^a^The obtained fish powder was added to the basal medium at different proportions

^b^The supernatant was used as a nutrient source for lipase production

A major concern associated with the use of fish wastes for microbial lipase production is the presence of lipids. A defatted fish meat preparation allowed a maximum lipase production (the optimum activity reached 14.20 U/ml while increasing the concentration of defatted fish meat) by *Staphylococcus epidermidis* CMST Pi 2 (Esakkiraj et al. 2010a). This observation confirmed the inhibitory effect of lipids on microorganism growth, as it was found when growing marine bacteria in fish peptone-based media (Vazquez et al. 2004) and *Staphylococcus xylosus* in tuna and sardine by-product-based media (Ben Rebah et al. 2008). In addition to that, regarding the effect of triglycerides on lipase production, cod liver oil showed as a suitable triglyceride to increase lipase production by *Staphylococcus epidermidis* CMST Pi 1 (Esakkiraj et al. 2010b).

Generally, microbial lipase production is highly influenced by medium components like nitrogen sources, carbon sources such as fatty acids, triglycerides and carbohydrates which can stimulate or repress lipase production. However, an adequate balance of nutrients and specific factors would ensure higher lipase activity (Ben Rebah et al. 2008; Ghorbel et al. 2005).

It appears, therefore, that there is a definite need for optimizing the microbial lipase production taking into consideration various factors especially the fish waste composition and microbial strain nutrient requirements. As pointed for proteases, the reported studies offer further exciting possibilities for the industrial use of the fish processing wastes.

## Chitinolytic enzymes

Chitinolytic enzymes are enzymes that degrade chitin and they are produced by various organisms such as viruses, bacteria, fungi, insects, higher plants and animals (Park et al. 1997). For example, these enzymes have been reported in several microorganisms such as *S. marcescens* (Suzuki et al. 2002), *Bacillus licheniformis* X-74 (Takayanagi et al. 1991), *Aeromonas* sp. No. 10S-24 (Ueda et al. 1995), *Streptomyces* sp. J. 13-3 (Okazaki et al. 1995), *Pseudomonas aeruginosa* K-187 (Wang and Chang 1997) and *Streptomyces griseus* HUT 6037 (Itoh et al. 2002). Recently, many novel chitinase/chitosanase producing microorganisms have been screened out from Taiwan soil (Wang et al. 2011).

Chitinolytic enzymes have various potential applications such as preparation of chitooligosaccharides and *N*-acetyl d-glucosamine which are known to have various biological activities (antimicrobial, antifungal, immunoenhancers, antitumor, etc.) (Tsai et al. 2000; Shen et al. 2009) with high interest in the pharmaceutical sector (Wen et al. 2002). Moreover, chitinases can be used for the control of pathogenic fungi in agriculture (Dahiya et al. 2005) and the degradation of crustacean chitinous waste in sea food industry. These enzymes are also useful for the preparation of single-cell protein, the isolation of protoplasts from fungi and yeast, etc. (Dahiya et al. 2006).

Microbial chitinase has been produced by liquid fermentation processes (batch, continuous and fed-batch fermentation) and is commercially available at a high cost (Dahiya et al. 2006). Generally, the production is controlled by physical factors (aeration, pH, and incubation temperature) and by the growth media components (Miyashita et al. 1991).

In order to increase the supply of active chitinase, it is necessary to reduce the production cost by using wastes for microbial growth. In this perspective, various chitinous materials from marine sources [shrimp shell powder (SSP), squid pen powder (SPP), shrimp and crab shell powder (SCSP)] have been utilized for chitinolytic enzyme production (Table 5) as alternative to waste disposal.

**Table 5:** Microbial production of chitinolytic enzymes using chitinous materials

Chitinous materials (rate added to the basal medium in % w/v)	Microbial strains	Enzyme type	Enzyme activity (U/mL)	References
Shrimp shell powder (SSP)				
0.5	*Pseudomonas* sp. TKU015	Chitosanase	0.026	Wang et al. (2008e)
0.5	*Pseudomonas* sp. TKU015	Chitinase	0.011	Wang et al. (2008e)
1	*Bacillus cereus* TKU018	Chitosanase	0.020	Wang et al. (2009d)
1.5	*Bacillus subtilis* TKU007	Chitosanase	0.030	Wang and Yeh (2008)
2	*Bacillus cereus* TKU006	Chitinase	0.089	Wang et al. (2009b)
1.5	*Serratia ureilytica* TKU013	Chitinase	0.032	Wang et al. 2009a
0.5	*Serratia* sp. TKU020	Chitinase	0.180	Wang et al. (2009c)
0.5	*Serratia* sp. TKU020	Chitosanase	0.400	Wang et al. (2009c)
1	*Serratia* sp. TKU016	Chitosanase	0.022	Wang et al. (2010)
Squid pen powder (SPP)				
3	Bacillus sp. TKU004	Chitosanase	0.14–0.16	Wang et al. (2009e)
1.5	*Serratia ureilytica* TKU013	Chitinase	0.037	Wang et al. (2009a)
2	*Serratia marcescens* TKU011	Chitosanase	0.030	Wang et al. (2008d)
0.5	*Serratia* sp. TKU020	Chitosanase	0.420	Wang et al. (2009c)
1	*Serratia* sp. TKU016	Chitosanase	0.008	Wang et al. (2010)
Shrimp and crab shell powder (SCSP)				
3	*Pseudomonas aeruginosa* K-187	Chitinase	0.650	Wang and Chang (1997)
2	*Bacillus amyloliquefaciens* V656	Chitinase	0.017	Wang et al. (2002a)
2	*Bacillus cereus* YQ308	Chitinase	1.400	Chang et al. (2003)
3	*Bacillus subtilis* W-118	Chitinase	4.200	Wang et al. (2006b)
1	*Monascus purpureus* CCRC31499	Chitinase	0.080	Wang et al. (2002b)

Dried materials of SPP SSP was prepared as described earlier (Wang et al. 2002a)

Interestingly, using shellfish chitin-containing wastes as the sole carbon/nitrogen source for chitinase/protease-producing strains can be exploited as potential biocontrol agents (Wang et al. 2006b). For example, SCSP of marine wastes induce the production of antimicrobial chitinases by *P. aeruginosa* K-187 (Wang and Chang 1997), *B. amyloliquefaciens* V656 (Wang et al. 2002a), *B. cereus* YQ308 (Chang et al. 2003), *B. subtilis* W-118 (Wang et al. 2006b) and *M. purpureus* CCRC31499 (Wang et al. 2002b). Moreover, *P. aeruginosa* K-187 isolated from the soil using SCSP as sole C/N source showed two kinds of bifunctional chitinases/lysozymes having antibacterial and cell lysis activities against many bacterial species (Wang et al. 1995; Wang and Chang 1997).

Silage obtained by lactic acid fermentation of shrimp head wastes containing chitin, proteins lipids and minerals was also used as substrate and inducer of β-*N*-acetylhexosaminidases of *Verticillium lecanii* in submerged fermentations (SF) and solid-state fermentations (SSF) (Shirai et al. 2001). The addition of sucrose or sugar cane pith bagasse reduces the growth time of *V. lecanii* (Matsumoto et al. 2001, 2004). Interestingly, a mixture of shrimp waste silage and sugar cane pith bagasse in SSF improved significantly the enzyme yield (Matsumoto et al. 2004). Chitinous materials from marine sources can be considered as good inducers for microbial chitinase production.

## Ligninolytic enzymes

Lignin is the most abundant natural aromatic polymer on earth and degradation of this recalcitrant aromatic polymer is caused by ligninolytic system from natural process in plants, animals, fungi and bacteria (Kirk and Farrell 1987). The ligninolytic system is an extracellular enzymatic complex including peroxidases, laccases and oxidases (Ruiz-Duenas and Martinez 2009). Ligninolytic enzymes have capacities to remove xenobiotic substances (such as hydrocarbons, phenols, perchloroethylene, azo dyes, carbon tetrachloride aromatics, pesticides, lignin, humic substances, etc.) which are introduced into the environment by numerous industrial activities. The presence of these xenobiotics in the environment can pose dangerous and unstable situations, because of their possible harmful effects on many organisms (Danzo 1997; Birnbaum 1994). Therefore, ligninolytic enzymes have potential applications in various sectors such as chemical, fuel, food, agricultural, paper, textile, cosmetic industrial sectors and in bioremediation purposes (Rodríguez and Toca 2006).

Generally, ligninolytic enzymes exhibit differential characteristics depending on various factors such as species, strains and culture conditions (Kirk and Farrell 1987). However, their production is very expensive and controlled by the raw material used for growth (Hacking 1987). In this perspective, various wastes such as apple pomace (Vendruscolo et al. 2008; Gassara et al. 2010), brewery by-product (Bartolome et al. 2003; Gassara et al. 2010), municipal and industrial sludge (Gassara et al. 2010) were used to produce ligninolytic enzymes (pectinase, lignin peroxidase, manganese peroxidase, cellulase and xylanase). The only study reporting the production of ligninolytic enzymes using fishery waste was that of Gassara et al. (2010). In this study, the production of lignin peroxidase, manganese peroxidase and laccase by *Phanerochaete chrysosporium* BKM-F-1767 was investigated in fishery residues (from SAUMMOM Inc., Montreal, Canada) by solid-state cultures (Table 6). Compared to apple wastes, the poor results of *P. chrysosporium* in fishery residues may be related to the unavailability of nutrients and the absence of cellulose in these residues (Gassara et al. 2010). The study of Gassara et al. (2010) on the production of ligninolytic enzymes using fishery residues might offer an impetus for further research in this field. Nevertheless, other microbial strains should be tested using various fish processing wastes as growth media taking into consideration various factors (waste composition, the microbial strains, the strain nutrient requirements, fermentation parameters, etc.).

**Table 6:** Maximum enzyme activity during growth (expressed in units/gram dry substrate) by solid-state cultures of *Phanerochaete chrysosporium* BKM-F-1767 in fishery residues compared to apple waste with and without inducer (Gassara et al. 2010)

Enzymes	Without inducer	With veratryl alcohol	With copper sulphate
Fishery residues			
Manganese peroxidase	47.4	17	17.4
Lignin peroxidase	nd	nd	nd
Laccase	nd	nd	94.4
Apple waste			
Manganese peroxidase	243.7	631.25	213.5
Lignin peroxidase	nd	nd	nd
Laccase	nd	141.4	719.9

*nd* not detectable

## Conclusions

The disposal of wastes generated by fishery processing industries represents an increasing environmental and health problem. However, these by-products have attracted considerable attention as an alternative feedstock and energy source, since they are abundantly available. Various microbes are capable of using these substances as carbon and energy sources beneficial in enzyme production process. A number of such substrates have been tested for the cultivation of microorganisms to produce several enzymes (protease, lipase, chitinases, peroxidases, laccases, oxidases, etc.). This may have numerous advantages for enzyme production process, such as superior productivity, simpler techniques, reduced energy requirements and reduced production costs. Generally, fish waste pre-treatments may be necessary to maximize microbial growth and enzyme production. However, each microbial strain has its own special conditions for maximum enzyme production. Therefore, it is of great significance to optimize the medium composition, taking into consideration the variability of fish waste composition, the nutrient requirements of microbial strain and fermentation parameters (pH, temperature, aeration, agitation, etc.). Nevertheless, the improvements in fish waste technology (pre-treatments, characterization, formulation, etc.) are still necessary before large-scale application of this new strategy can be realized.
